# Exploring Academic Emotions Using Design Thinking Applied to Elementary School Learning Stress Adaptation

**DOI:** 10.3390/healthcare12212103

**Published:** 2024-10-22

**Authors:** Fang-Suey Lin, Gui-Shu Chen

**Affiliations:** Graduate School of Design, National Yunlin University of Science and Technology, Yunlin 64002, Taiwan; linfs@yuntech.edu.tw

**Keywords:** design thinking, academic emotions, stress adaptation courses, health education, student mental health

## Abstract

Background: Students’ learning stress adaptation is crucial. Research on design thinking in stress adaptation and academic emotions is still lacking. Methods: This study developed “Stress Relief Design” instructional activities using a mixed-method research design, including student academic emotion journals and a stress relief agreement survey for data collection. This study was approved by the Human Research Ethics Committee and conducted in accordance with ethical guidelines. Participants were 24 students, aged 10 to 12, recruited through open enrollment. The instructional activities were conducted in a holiday workshop format, consisting of fifteen sessions over 5 days, with three sessions per day. Results: The results indicated that students predominantly experienced positive academic emotions (joy, pride, hope, and relaxation), with negative emotions (anxiety and boredom) being less common. Students reported that the course helped them relieve psychological stress. Conclusions: Therefore, design thinking is suitable for application in stress adaption courses and creates a learning environment that supports students’ academic emotions, making it a critical learning focus for modern students. This study contributes to the academic research on the application of design thinking in elementary school health education and learning stress adaptation, as well as on academic emotions.

## 1. Introduction

In 2023, both the United States and the European Union highlighted the mental health crisis faced by children and adolescents. In the U.S., over 10% of adolescents suffer from depression, significantly impairing their abilities in school, work, family interactions, and social life [1]. In the European Union, approximately 11.2 million (13%) children and adolescents aged 19 and below experience mental health issues [2]. In Taiwan, the severity of stress among junior and senior high school students exceeds a significant level, at 12.2%, with the top three sources of stress being schoolwork (76.9%), future prospects (67.3%), and social relationships (43.0%) [3]. The World Health Organization (WHO) designates October 10th as “World Mental Health Day” each year, with the theme for 2023 being “Mental health is a universal human right”, and calls for increased awareness and action towards mental health to ensure that everyone has access to quality mental healthcare (2023) [4].

It has been demonstrated that academic stress has a negative impact on students’ mental health [5,6]. Excessive psychological stress in children can negatively impact their working abilities, leading to reduced academic performance across various subjects [7]. Methods to help children or students adapt to stress are gaining attention, such as mindfulness training and reading books as intervention strategies [8,9]. However, a 2016 study on stress in American students aged 7 to 11 revealed that students are generally underprepared to handle everyday stress through socially appropriate and effective coping strategies [10]. Promoting mental health includes educating students on how to adjust and alleviate stress effectively, which is an important educational objective. “Positive attitude and emotion, as well as stress management skills” are specified as learning objectives in the health and physical education curriculum for elementary schools in Taiwan [11]. Health education, defined as systematic social activity aimed at helping target populations or individuals improve health-related behaviors, ultimately aims to maintain health status by changing the mindset of the target. Health education may encompass individual education and development, as well as mass media information and education [12].

**Figure 1:**
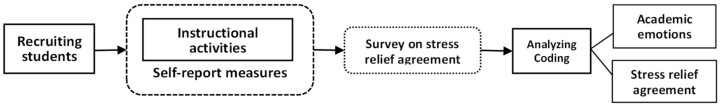
Study procedures.

The Hasso Plattner Institute of Design at Stanford University (commonly known as d. school) proposes the design thinking process in five steps: Empathize, Define, Ideate, Prototype, and Test (Figure 1). While these five steps may appear linear, design thinking in practice is an iterative process, where encountering issues allows for revisiting previous steps for reiteration [13,14,15]. In recent decades, design thinking has been widely discussed and applied in many fields. After applying design thinking in classroom teaching, it was found to effectively enhance the group learning atmosphere, stimulate creativity, and enhance self-creative identity among students [16]. The combination of design thinking and gamification methods applied in the production of health education videos not only helped solve complex issues between healthcare professionals and video producers but also promoted communication among students from different professional backgrounds [17]. Design thinking has also been applied in the healthcare sector to assist professionals in acquiring new knowledge, fostering innovative applications, and establishing mechanisms to address the complex challenges faced by society and the healthcare industry [18]. Research has shown that applying design thinking in healthcare education not only boosts students’ creative confidence but also provides innovative approaches and thinking for healthcare issues, particularly in nursing education, fostering more empathetic interactions between nurses and patients [19]. In the realm of mental health-related research, studies have noted that stress and burnout affect all health professions from education to practice, including patients, students, trainees, and healthcare professionals. This research utilizes design thinking and systems thinking to formulate strategies for addressing stress and burnout, and improving the well-being of students, interns, teachers, and healthcare professionals [20]. According to the study by Lin and Chen (2024) on the application of design thinking in stress adaptation courses within the field of health and physical education, it was found that after participating in the instructional activities, elementary school students experienced a significant improvement in their self-efficacy related to understanding various sources of stress, different stress-relief methods, and the effectiveness of those methods. In terms of empathy, students became more confident in their ability to understand and empathize with the stress and emotions of their classmates or family members. They could better identify whether their peers or family members were experiencing stress and became more adept at understanding and caring for them, as well as using methods to determine the sources of their stress. Regarding the evaluation of the design thinking-based curriculum, students reported that it provided a positive learning experience and helped them recognize the creativity of others [21].

A study on the effects of the mindfulness-based program “Compassion and Attention in Schools” (Compas), conducted with 5th, 7th, and 8th grade students, showed significant improvements in effortful control, school well-being, and peer relationship perception in the experimental group compared to the control group [22]. Another study examined the feasibility and effectiveness of the Compassionate Mind Training for Teachers (CMT-T) program on psychological and physiological health indicators. Participants completed self-report measures over an 8-week period. The results demonstrated that CMT-T, as a compassion-centered intervention, enhanced teachers’ compassion and well-being, and reduced psychophysiological distress [23]. The first step in design thinking is Empathize, and while the meanings of empathy and compassion are not exactly the same, this study aims to explore whether the design thinking model can help students alleviate stress.

On the other hand, emotions have a significant impact on learning. Emotions are considered crucial for students’ academic learning, achievement, personality development, and well-being [24,25,26]. In contemporary emotion research, emotions are defined as multidimensional phenomena involving a series of coordinated psychological processes, including affective, cognitive, physiological, motivational, and expressive components [26,27]. Academic emotions refer to the emotions experienced by students during learning activities [24,26], which can either facilitate or hinder learning. These emotions include positive feelings such as curiosity and happiness, as well as negative feelings such as frustration and anxiety; they contribute to achievement and personal growth. Experiencing positive emotions can help students envision goals, promote creative problem-solving, and support self-regulation. Conversely, excessive negative emotions towards learning and exams can impact academic performance, leading to dropout and negative effects on health. The profound impact of emotional experiences may also be reflected in the annual number of student self-harm incidents [28,29].

Pekrun described the Control-Value Theory of Achievement Emotions and its implications for educational research and practice. It is based on the premise that the assessment of control and value is central to the arousal of achievement emotions, including activity-related emotions such as enjoyment, frustration, and boredom experienced during learning, as well as outcome emotions such as joy, hope, pride, and anxiety related to success or failure, despair, shame, and anger [30]. Academic emotions are classified into positive activating (joy, hope, and pride), positive deactivating (relief and relaxation), negative activating (anger, anxiety, and shame), and negative deactivating (despair and boredom). Additionally, emotions can be grouped based on their object focus. Specifically, regarding the role of emotions in students’ academic engagement, object focus is crucial as it determines whether emotions are relevant to the current academic task. Academic emotions can be further categorized based on object focus into Achievement Emotions related to success or failure in exams or learning outcomes, Epistemic Emotions related to learning cognitive processes, Topic Emotions related to learning materials, and Social Emotions related to interactions with others in learning activities [26].

Design-Based Learning (DBL), along with learning through and by design, includes approaches that apply design thinking principles within problem- or project-based learning environments. Generally, DBL involves open-ended exploration, learning from trial and error, reflection, teamwork, and supporting tools [31]. Research on DBL and student emotions often employs four common assessment methods, surveys, interviews, observations, and video coding, often combining several methods. Quasi-experimental designs have also been used to compare the performance of DBL and non-DBL students [32]. Participants in these studies range from elementary to university students, with sample sizes from nine to over a thousand students, covering a broad spectrum.

The research focuses on various aspects, including learning activities, the teacher’s role, materials and resources, and group dynamics. A case study aimed to gain deeper insights into children’s emotional experiences during DBL, involving nine children aged 12–13. This study assessed emotions using non-verbal self-report tools (emotion cards) and verbal tools (Geneva Emotion Wheel questionnaire) [31]. Additionally, the same researchers developed EmoForm, a retrospective self-report tool based on the curriculum, to capture children’s emotional changes over time during DBL, considering the potential interference of assessment tools with academic emotions [33].

Despite accumulating evidence of the benefits of DBL on student emotions—such as excitement, satisfaction, pride, enjoyment, enthusiasm, curiosity, happiness, relaxation, engagement, self-efficacy, positive attitudes towards teachers, and increased interest in science—many questions remain unanswered [32].

Referencing research conclusions that compassion can alleviate stress, theories of academic emotions, and the foundational studies of DBL—which suggest that design-based instructional models can stimulate positive emotions in students—this study aims to explore whether applying the design thinking model in a stress adaptation course can elicit positive academic emotions in students and help them achieve stress relief.

The motivation behind this study stems from the extensive time students spend learning in school, the abundance and heaviness of learning content, the multitude of stressors students face, and their impact on mental health. It is crucial for students to learn how to recognize and alleviate stress. There is limited research on students’ psychological well-being in schools, especially regarding elementary school students. Therefore, this study aims to explore elementary school students’ academic emotions through the lens of design thinking and address the following research questions:

(1) What are the academic emotions of students in “Design Thinking Model Applied in Stress Adaptation Course”? What are the causes of these emotions?
(2) Does “Design Thinking Model Applied in Stress Adaptation Course” help students relieve stress? What are the reasons for this?

## 2. Methods

### 2.1. Ethics Approval

This study was approved by the Human Research Ethics Committee of National Chung Cheng University (No. CCUREC112120401) on 29 January 2024. The data obtained in this study were stored electronically in an encrypted folder, and the content of the data analyzed had been de-identified. The data were used solely for the purpose of academic research and publication and were not employed for any other purposes.

### 2.2. Participants

In this study, we recruited fifth- and sixth-grade students from a public elementary school in central Taiwan through open enrollment. Participants were required to have their informed consent forms filled out and submitted by themselves and their parents. The actual participants consisted of 10 boys and 14 girls, totaling 24 individuals, aged between 10 and 12 years old. They were divided into seven groups, with each group comprising three to four students from different classes, none of whom had prior experience with design thinking. The study was conducted during the winter vacation period from January to February 2024 and was facilitated by a teacher with experience in design thinking instruction, who also served as the researcher. The teacher has 20 years of teaching experience at the elementary school level and has been teaching at this particular school for 10 years, making them a familiar figure to the students.

### 2.3. Study Design and Study Procedures

In this study, we constructed a five-day instructional program applying design thinking to stress adaptation learning. A total of 24 students voluntarily participated in this study, recruited through an open call. This study employed a mixed-method research design [34], which involves first collecting qualitative data and using qualitative analysis methods to obtain qualitative findings (such as identifying key themes). The qualitative data is then converted into quantitative data for further quantitative analysis, leading to quantitative conclusions. The findings from both approaches are subsequently integrated. Regarding data collection, this study employed self-report measures [31] to gather information on students’ academic emotions. At the end of each day’s session, students were asked to write academic emotion journals. Upon completion of the instructional activities, students were surveyed on their level of agreement, with reasons, regarding the specific question. Finally, the qualitative data from these academic emotion journals and the responses of the specific question were analyzed, coded by the researchers, and subsequently transformed into quantitative data (Figure 1).

### 2.4. The “Stress Relief Design” Instructional Activities

**Figure 2:**
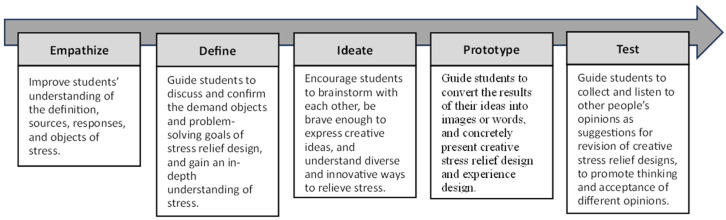
Instructional objectives of the “Stress Relief Design” instructional activities.

Based on the content of the “Stress Adaptation” curriculum in the elementary school health and physical education textbook, this study applied the design thinking five-step process—Empathize, Define, Ideate, Prototype, and Test—as the instructional framework to design activities centered around “stress relief design”. The instructional objectives are presented in Figure 2. The study was conducted in the form of a holiday workshop, with three sessions per day, each lasting 40 min, for a total of five days comprising 15 sessions, conducted in group settings. During the course, students wrote academic emotion journals, and after the activities, they were asked to answer the specific question: “I feel that participating in the stress relief design activities can help me relieve stress”.

The teacher, also the researcher, designed two types of worksheets: an interview record worksheet and a group learning worksheet, to guide students in achieving the instructional objectives and serve as learning tools for recording and discussion during the learning process. Worksheets were frequently utilized in student course activities, serving not only as learning tools but also as records of learning outcomes after activities, facilitating teachers in reviewing and assessing students’ learning progress and achievements. Student learning records and academic emotion journals are presented in Table 1 and the teaching procedures are listed in Table 2.

### 2.5. Measures

One of the advantages of self-report measures is their ability to help collect emotional data that cannot be directly observed. Researchers can obtain such data from respondents at a relatively low cost, using methods such as paper-and-pencil rating scales, questionnaires, and interviews [31]. In this study, students used the notebooks we provided to write academic emotion journals after the daily class session. Students were asked to write about their emotions, feelings, thoughts, and the reasons behind them during the learning activities. The teacher encouraged students to write freely with no word limit. All students’ notebooks were collected after the completion of all instructional activities (Table 1), and the content of the notebooks was transcribed verbatim to facilitate coding and analysis.

In addition, students were required to answer a specific question: “I feel that participating in the stress relief design activities can help me relieve stress”, which was developed based on the research results of Lin and Chen (2024) [21]. This question was presented using a five-point Likert scale to assess their level of agreement. Additionally, students were asked to provide a written explanation of their reasons to determine whether the instructional activity effectively helped them relieve stress.

### 2.6. Content Analysis

#### 2.6.1. Academic Emotion Journals

This study conducted content analysis on data derived from students’ academic emotion journals and responses to open-ended questions, and employed MAXQDA2022 for coding and classification. Researchers were tasked with selecting codes and conducting thematic and narrative coding analysis using a classification directory. This study referenced the descriptions of academic emotions by Pekrun and Linnenbrink-Garcia (2012) and Lai and Wu (2016) [26,35] as the classification directory, as presented in Table 3. The coding classification directory comprised seven academic emotions: anger, anxiety, boredom, enjoyment, hope, pride, and relaxation. Additionally, these emotions were further categorized into positive activating emotions (joy, hope, and pride), positive deactivating emotion (relaxation), negative activating emotions (anger and anxiety), and negative deactivating emotion (boredom). Two coders were employed for coding, ensuring consistency. The coding principle is explained as follows: (S5(2)-91), where “S” represents “student”, “5” represents the group number, “(2)” represents the student number, and “91” represents the paragraph sequence.

#### 2.6.2. The Specific Question About Stress Relief

On one hand, we statistically analyzed the Likert scale responses to this specific question to present the distribution of agreement levels among all students. On the other hand, the written explanations provided by the students were transcribed verbatim. These transcripts were then read repeatedly, with key sentences and paragraphs highlighted, and keywords identified based on the students’ wording. After forming concepts around these keywords, they were grouped into themes. The coding principle is explained using the notation (Q-S3(1)-1), where “Q” represents this question item, “S” stands for “student”, “3” denotes the group number, “(1)” indicates the student number, and “1” represents the sentence order.

## 3. Results

### 3.1. Academic Emotions

The content of all academic emotion journals included one hundred ninety-three entries, consisting of 8038 words, excluding nine unrelated entries. These journals were written by 24 students. The emotional expressions in the entries were coded by the researchers through mutual judgment. Some entries explicitly described emotions with words like “fun”, “very interesting”, or “felt bored”. Other entries conveyed emotions through the meaning of the text, such as “I believe we will do even better!” which expresses feelings of “hope and confidence”. Most students participating in the course experienced enjoyment, some felt pride, hope, or relaxation, while a few students experienced anxiety, anger, or boredom.

**Figure 3:**
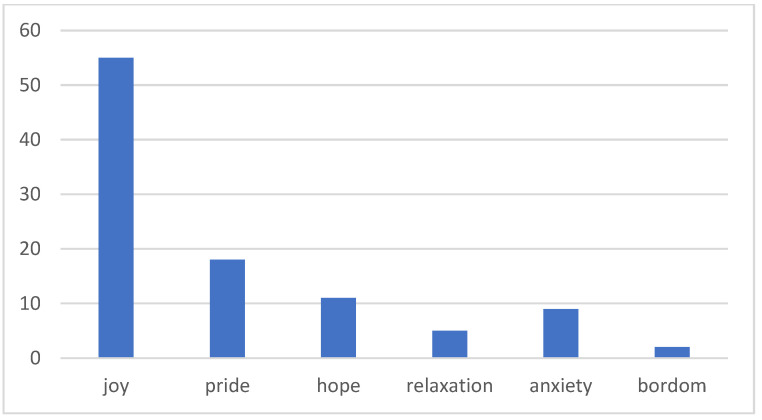
Proportion of academic emotions occurrence.

**Figure 4:**
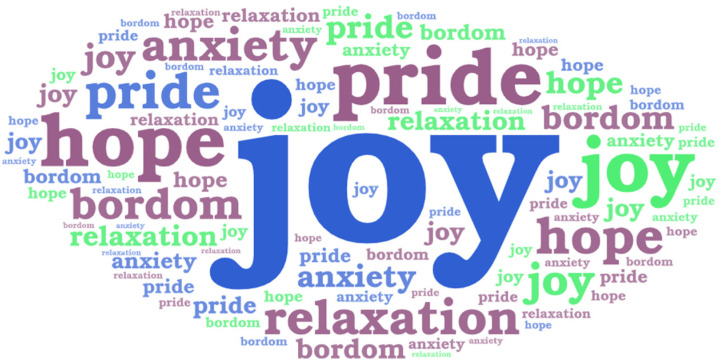
Word cloud of academic emotions occurrence.

Based on the coding results, six types of academic emotions were identified (Table 4), namely joy (55%), pride (18%), hope (11%), relaxation (5%), anxiety (9%), and boredom (2%) (Figure 3 and Figure 4). Among these, joy, pride, and hope are classified as positive activating emotions, accounting for 84%; relaxation is classified as a positive deactivating emotion, accounting for 5%; anxiety is classified as a negative activating emotion, accounting for 9%; and boredom is classified as a negative deactivating emotion, accounting for 2%. Positive emotions account for 89%, while negative emotions account for 11%. The results of this study indicate that students primarily experience positive emotions during stress relief design activities.

### 3.2. Causes of Academic Emotions

Most students clearly articulated the reasons behind their academic emotions. At the beginning of the “Stress Relief Design” activities, a few students found the teacher’s lecture-based teaching to be boring. During the five-step design thinking activities, many students encountered various unknown challenges, such as empathizing (interviewing others), defining problems, brainstorming ideas, designing prototypes, and presenting their group’s designs to the entire class. Initially, they felt nervous, but after overcoming these challenges, they experienced pride and a sense of accomplishment. The opportunity to freely express their ideas, which is rare in other courses, made the students feel happy. These novel experiences also made them feel intrigued and hopeful. The theme of “stress relief design” allowed students to release their stress effectively through discussion, sharing, and empathy within their groups, achieving the desired stress-relief effect. Throughout these challenges, the attitudes of group members and the teacher played a critical role in shaping the students’ overall academic emotions.

**Figure 5:**
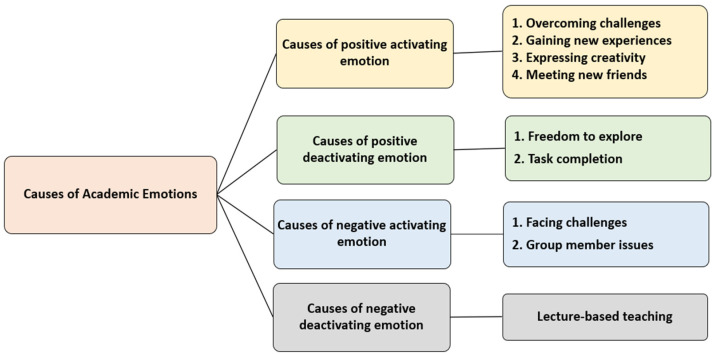
Causes of academic emotions.

A careful analysis of the causes of academic emotions (Figure 5, Table 5) reveals that positive activating emotions stem from overcoming challenges, gaining new experiences, expressing creativity, and meeting new friends. Positive deactivating emotions result from freedom to explore and task completion. Negative activating emotions arise from facing challenges and group member issues. Negative deactivating emotions are attributed to lecture-based teaching. Based on the aforementioned reasons, a further analysis and categorization of academic emotions can be classified into achievement emotions (overcoming challenges), cognitive emotions (gaining new experiences and expressing creativity), and social emotions (meeting new friends and group member issues) [26].

### 3.3. Survey on Stress Relief Levels of Agreement

**Figure 6:**
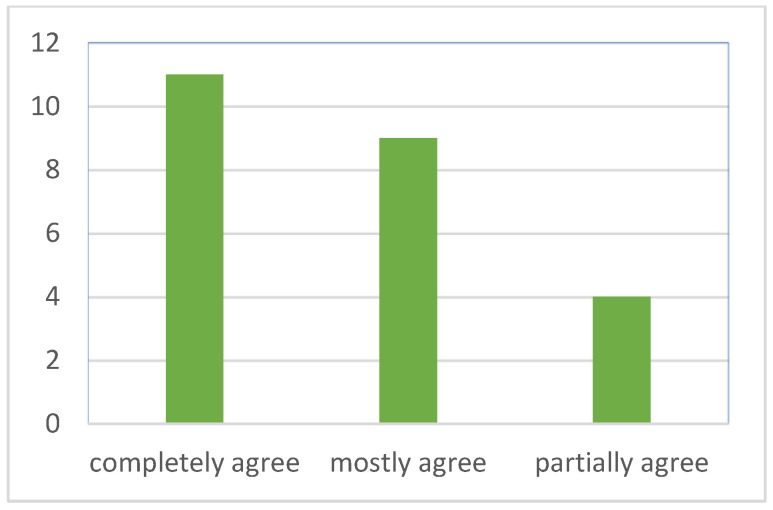
Number of participants and their levels of agreement on the effectiveness of stress relief design activities.

The survey results for the specific question, “I feel that joining the stress relief design activities can help me relieve stress”, are divided into two parts. The first part presents the Likert scale measuring the level of agreement. The statistical results show that among the twenty-four students, eleven students indicated “completely agree”, nine students indicated “mostly agree”, and four students indicated “partially agree” (Figure 6). This indicates a high level of agreement among students that the activity helps relieve stress.

**Figure 7:**
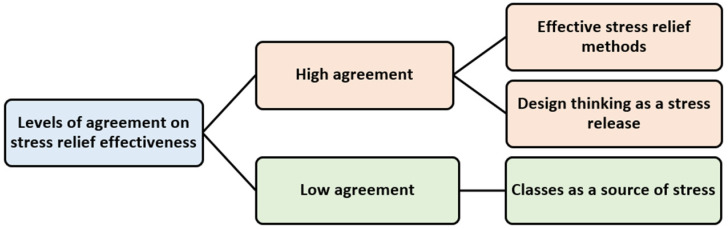
Reasons for differences in levels of agreement on stress relief effectiveness.

The second part involves the reasons behind the students’ varying levels of agreement. The responses included twenty entries, consisting of 492 words, excluding four unrelated entries. After transcription, analysis, and coding, the reasons for high agreement were identified as “effective stress relief methods” and “design thinking as a stress release”. The reason for low agreement was identified as “classes as a source of stress” (Table 6, Figure 7).

From the above analysis of reasons, it can be inferred that the tasks inherent in Stress Relief Design activities essentially facilitate stress relief effects. After individual stress is alleviated, there are better interactions with family members. However, the dynamics among group members have a crucial impact on emotions and stress relief situations.

## 4. Discussion

### 4.1. Exploring the Causes of the Impact of Design Thinking Curriculum on Students’ Stress Management and Academic Emotions

The aim of this study is to understand the academic emotions and their underlying causes when students participate in a design thinking applied Stress Adaptation course, as well as whether such a course can help students relieve stress. The research findings indicate that students primarily experience positive emotions during “Stress Relief Design” activities, with fewer instances of negative emotions, but the causes of both warrant further exploration. These results corroborate findings from other studies [16,21,31]. A closer examination of the reasons behind academic emotions reveals three main points. Firstly, most students enjoy and accept overcoming challenges, gaining new experiences, and exercising creativity. Since the essence of design thinking encourages students to express their ideas and acknowledges various forms of creativity, despite experiencing negative emotions such as nervousness during challenges, they often transform into positive emotions like pride and a sense of achievement after completing tasks. Thus, assisting students in transforming negative emotions into positive ones can significantly benefit their self-awareness. Secondly, as design thinking emphasizes group interaction and communication, where members with different traits and expertise learn from each other through frequent discussions, generating innovative ideas [17,19], the interaction among group members critically influences students’ academic emotions. Elementary school students are still learning how to interact with others, and they may easily experience negative emotions when faced with disagreements or conflicts among group members, which may not be easily resolved in a short period. Such situations have always been a significant challenge in classroom teaching. Thirdly, different teaching methods significantly affect academic emotions. For example, when teachers adopt a lecture-based approach, academic emotions tend to be calm or even boring. Hence, the appropriate use of teaching methods can evoke different academic emotions. Emotions influence various cognitive processes that contribute to learning, such as perception, attention, social judgments, cognitive problem-solving, decision-making, and memory processes. As suggested by Pekrun, in the emotional climate of the classroom, we need to design learning environments that are suitable for students’ emotions and test the effectiveness of these environments [26].

In the part of the survey on levels of agreement on the impact of participating in stress relief design activities on stress relief, students agreed that this activity could be helpful for stress relief. Comparing the reasons provided by students with the reasons for the emergence of positive emotions, there are some similarities between the two. For example, design thinking as a teaching method is more enjoyable and relaxing. Furthermore, the course theme focused on stress adaptation, and students reported having learned effective stress relief techniques. Some even noted improved interactions with family members as a result, indicating the course’s effectiveness and its potential to enhance students’ ability to manage stress [10,21]. On the other hand, some students felt stressed and unable to relax because they were required to write learning sheets as part of the course or were asked to write academic emotion journals for research purposes. The interaction among group members also had a critical impact on the stress relief levels of agreement, and teachers must effectively guide and counsel students to prevent the escalation of negative emotions. According to the research findings, the application of design thinking in stress adaptation courses can facilitate stress relief for students. Based on the research findings, it is conjectured that academic emotions are correlated with stress and mental health [5,6], warranting continued research in the future.

This study has several limitations that should be considered when interpreting the results. First, the study was conducted during the holiday period, meaning students were not directly affected by academic pressure from school. Since academic pressure is typically one of the primary sources of stress for elementary school students [3,5,6], conducting the intervention in a context where students were not experiencing this type of pressure may limit the comprehensive understanding of their academic emotions and the degree to which they perceive stress relief. Future research should consider conducting interventions during the school term to more accurately assess the effects of design thinking on stress relief when facing academic pressure.

Second, the design and implementation of the program lasted only five days, which is relatively short and may be insufficient to observe comprehensive changes in student stress levels. The long-term stress adaptation abilities of students and the lasting effects of design thinking cannot be fully captured within such a short intervention period. Therefore, the generalizability of the findings is constrained by the time limitation, as they do not reflect the changes and adaptation processes in student stress over a longer period. Future studies should extend the intervention duration and use longer-term follow-up assessments to more comprehensively observe the long-term impact of the “Stress Relief Design” activities on stress relief.

Lastly, this study lacked a control group, making it difficult to determine whether changes in students’ emotions and stress adaptation were solely attributable to the “Stress Relief Design” activities. External factors such as the holiday period, other activities, or environmental changes were not controlled for, making it challenging to establish causality. Future research should include a control group and adopt a randomized design to more effectively compare differences between the experimental and control groups.

### 4.2. A Review of Data Collection Methods on the Reliability and Validity of Academic Emotional Data

In selecting the data collection method, we aimed to minimize interruptions to the students’ learning experience. Therefore, we asked students to write their academic emotion journals only after the conclusion of each day’s course. The research results indicate that this approach achieved the desired effect, with only a very small number of students experiencing negative emotions due to writing the journals [31]. Additionally, the teacher continuously encouraged students to freely express themselves and supported all student ideas, even dissenting opinions. This approach positively impacted the credibility and reliability of the emotional data, as it helped prevent students from expressing only positive emotions or opinions [31]. However, since students gathered in groups and could easily share opinions, this might have had a slight negative impact on the credibility and reliability of the emotional data. This is an aspect that future research should consider. Since the self-report writing of academic emotions is a subjective expression, related research has incorporated objective data, such as facial expression analysis from classroom video recordings, as part of a triangulation method [32].

## 5. Conclusions

Emotions influence various facets of learning. Creating a learning environment that enhances positive academic emotions for students is crucial for maintaining their psychological well-being. This study applies design thinking to stress adaptation courses in health education to explore students’ academic emotions and stress relief levels of agreement during the learning process. The research findings reveal that the application of design thinking in stress adaptation courses contributes to enhancing positive academic emotions and facilitating stress relief among students. The generation of positive academic emotions is complex and multifaceted, including experiencing overcoming challenges, acquiring new experiences, fostering creativity, and establishing good interactions with new friends and group members within the five steps of design thinking. A few factors contributing to negative academic emotions include conflicts among group members and the use of lecture-based teaching by instructors. Additionally, students believe that learning stress adaptation through design thinking helps alleviate stress. This study provides valuable insights into design thinking, academic emotions, and stress adaptation learning, aiding in the design of learning environments that cater to students’ academic emotions and the formulation of effective educational measures to maintain students’ psychological well-being.

## 6. Limitations of the Study

The limitations of this study are as follows. Firstly, the analysis of academic emotions and their causes in this study is based on the content of students’ post-class diaries, where there may be discrepancies between students’ written expressions and subjective emotions. Secondly, some students may conceal their true feelings due to certain factors, which may not be reflected in the written content. Thirdly, as this is an academic research project, it requires students to provide textual data, which may simultaneously interfere with students’ emotions. Future research could focus on how to more accurately obtain students’ academic emotions and thoughts regarding participation in teaching activities.

## Figures and Tables

**Table 1:** Academic emotion journals and learning records of participating students.

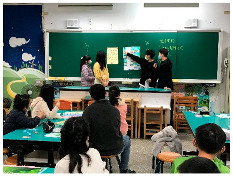 a. Step 2, Define: Students in groups reported and explained the problems and objects they wanted to solve to their classmates.	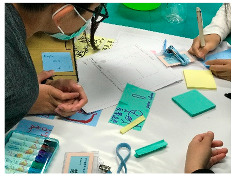 b. Step 4, Prototype: Students in groups expressed their ideas in the form of drawings and created prototypes.	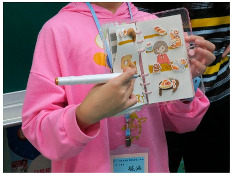 c. Step 5, Test: Students in groups showed and explained their prototype to their classmates to obtain their opinions.
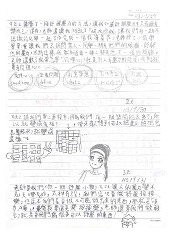 d. The academic emotion journal: This study provides notebooks for students to write freely after class.	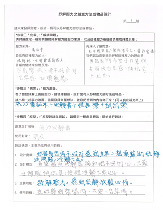 e. The group learning worksheet 1: The worksheet was designed based on the five steps of design thinking to guide students to develop creative design.	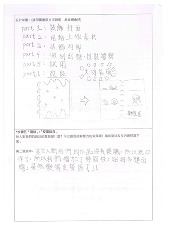 f. The group learning worksheet 2: Students presented the ideas generated in the Ideate step in the form of sketches.
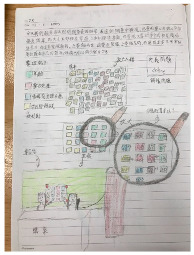 g. The academic emotion journal 1: This student expressed her gains and feelings in the form of words and drawings on the academic emotion journals.	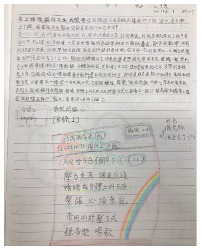 h. The academic emotion journal 2: Some students described the class process and academic emotions in details. For example: After interviewing others, this student were very happy to have gained a better understanding of stress.	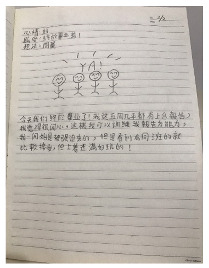 i. The academic emotion journal 3: Some students described their feelings relatively simply and concisely. For example: This student was very happy to go on stage and report.

**Table 2:** The procedures for the “Stress Relief Design” instructional activities.

Day/Session	Steps	Activities
Day 1, Sessions 1–2	Reading the textbook	Prior to the formal stress relief design teaching activity, the teacher guides students to read and comprehend the content of the “Stress Adaptation” unit in the textbook.
Day 1, Session 3	The “Empathize” step	The teacher leads students to understand the objectives of the activity, instructing them to utilize interview recording methods during their free time to investigate and understand the stress sources and emotional responses of others, and to complete interview record learning sheets during their free time.
Day 2, Sessions 4–6	The “Define” step	Students synthesize the data obtained from interviews, and set a scenario of stress sources and emotional responses, defining it as the target of stress relief design and completing the first part of the group learning sheet.
Day 3, Sessions 7–9	The “ Ideate “ step	Students learn creative ideation methods, engage in creative brainstorming, and use visualization tools such as posters, colored pens, and sticky notes to present their ideas through writing, drawing, etc., completing the second part of the group learning sheet.
Day 4, Sessions 10–11	The “Prototype” step	Students design items or methods that can relieve stress, presenting them through drawings and accompanying text, completing the second part of the group learning sheet.
Day 4, Session 12	The “Test” step (part one)	Groups present their stress relief design sketches and design concepts to the audience, along with the intended users, engaging in interaction and receiving feedback or further explanations about the purpose of their work, to determine whether modifications are needed.
Day 4, Sessions 13–14	The “Test” step (part two)	Groups present their revised work again and listen to feedback from others.
Day 5, Session 15	Conclusions	The stress relief design teaching activity concludes.

**Table 3:** Compilation of academic emotional classifications.

Coding Category	Similar Words from Diaries
anger	angry, disgusted
anxiety	anxiety, irritability, nervousness, worry
boredom	boring
joy	interesting, fun, interest, like, happy
hope	be confident, confident, expect
pride	a sense of accomplishment
relaxation	relax

**Table 4:** Coding results of academic emotions.

Parent Code	Child Code	Grandchild Code (If Applicable)	Content Examples
Positive activating emotion	Pride	Accomplishment	Today we improved Bubble Slime. I think our work was very good and we learned a lot. Thank you, Teacher! (S5(2)-91) (Test step) Today we have roughly designed some creative ways to relieve stress or the main items in item design. I think although my head is about to explode, it still feels good. (S4(3)-70) (Ideate step)
	Hope	Confidence	During the design presentation, although some people raised questions about our design, I believe we will do better! (S4(4)-77) (Test step) Today’s class was so fun! Although it failed, it was still very interesting. There is a saying: Failure is the mother of success. I believe we will do better. (S6(3)-112) (Prototype step)
	Joy	Interesting	The class was very fun, as if a detective was working on a case. We integrated the stress sources and stress relief methods of the interviewees and reported on the stage. It was very interesting, and it also gave me an extra stage experience. (S5(2)-88) (Presenting) Today’s class is the most fun of all the past few days, maybe because we are designing it ourselves today! (S7(2)-125) (Prototype step)
Positive deactivating emotion	Relaxation		Classes are very relaxing. (S2(4)-31) (Day 1) The teacher said that we would go on stage to give a report today. Our group did not know why we were so nervous, but as soon as we got on stage and finished speaking, I breathed a sigh of relief! (S2(2)-20) (Prototype step)
Negative activating emotion	Anxiety	Nervous	Yesterday it took us a long time to complete the report. We had spent so much effort on it, but when we took the stage, we were hesitant and unable to express ourselves. This made me embarrassed and sad. I am very introverted and do not know how to express the content of the report. I hope that the following courses can help solve the stress problem. (S1(2)-4) (Presenting) Feelings: Encountering difficulties with something invented. Thoughts: It feels very hard. Everyone racked their brains for buttons. Originally we were going to make Doraemon, but I did not have three scientists, so we had to make Stress Relief Buttons. (S2(3)-27) (Ideate step)
	Anger		Today someone kept saying that I think the balloon contains plasticizer, so I am sick, but they do not understand that plasticizer will enter the body and cause precocious puberty if it is eaten on the hand. In addition, plasticizers are not easy to wash off, so I am very unhappy. In the morning, I also discovered that someone always thought that I was saying bad things about him behind his back, but I clearly often heard him scolding me and saying bad things about me. (S6(1)-102) (Test step)
Negative deactivating emotion	Boredom		Although the process of introducing “Stress Adaptation” made me feel a bit bored, the process of group interaction was really interesting. (S1(3)-10) (Day 1)

**Table 5:** Coding results of causes of academic emotions.

Parent Code	Child Code	Content Examples
Causes of positive activating emotion	Overcoming challenges	When I went on stage to report today, I felt less nervous! I feel like I have made progress. My favorite moment today was when I was giving my presentation. I felt nervous before going on stage, and I also felt a great sense of accomplishment when people on stage and backstage laughed! The presentation was less tense this time. (S7(1)-119) We finally graduated! I have been on stage to report for almost five weeks, and I feel very happy that I can train my reporting skills. (S2(3)-28) In order to report on stage smoothly, our group practiced very hard after class, and finally successfully presented our work, although it was not as perfect as during practice. (S4(1)-57)
	Gaining new experiences	The class was very fun, as if a detective was working on a case. We integrated the stress sources and stress relief methods of the interviewees and reported on the stage. It was very interesting, and it also gave me an extra stage experience. (S5(2)-88) The teacher asked us to make a small stress relief object that can make people’s stress disappear. Oh my god, it is amazing! (S2(2)-19) Today is a lesson about in-depth understanding of problems and creative ideas. This class not only allows us to use our imagination, but also allows us to understand the problem in depth. It is really interesting. (S4(4)-75)
	Expressing creativity	Today we improved Bubble Slime. I think our work was very good and we learned a lot. Thank you, Teacher! (S5(2)-91) Today we have roughly designed some creative ways to relieve stress or the main items in item design. I think although my head is about to explode, it still feels good. (S4(3)-70) Today’s class is the most fun of all the past few days, maybe because we are designing it ourselves today! (S7(2)-125) It was very, very fun today. Our group published toys and put a lot of emoticon stickers on the report, which was very funny. Today was very fun. At first, everyone disagreed, but then the students in the same group changed it to a lottery machine, and finally everyone accepted it. (S3(1)-38)
	Meeting new friends	On the first day of class, I was very excited. I met a lot of people I knew. By the way, I got to know the person opposite me named Aquarius. I was very happy. (S5(3)-94) Today is the last day of the course on Design Thinking Application and Stress Adjustment. I think it is very meaningful that I got to know one more classmate in these few days, because I rarely talk to people I do not know. I think this is the most important thing in these five days. I was forced, but I felt good after finishing it. (S4(3)-72) I was excited and I met new people and I was happy. (S5(1)-80)
Causes of positive deactivating emotion	Freedom to explore	Classes are very relaxing. (S2(4)-31)
	Task completion	The teacher said that we would go on stage to give a report today. Our group did not know why we were so nervous, but as soon as we got on stage and finished speaking, I breathed a sigh of relief! (S2(2)-20)
Causes of negative activating emotion	Facing challenges	I am very nervous to go on stage to give a speech. Thoughts: Fortunately, everyone’s talk was interesting, which eased the embarrassment. (S2(3)-25) Yesterday it took us a long time to complete the report. We had spent so much effort on it, but when we took the stage, we were hesitant and unable to express ourselves. This made me embarrassed and sad. I am very introverted and do not know how to express the content of the report. I hope that the following courses can help solve the stress problem. (S1(2)-4) When I went on stage to report, I was shaking. (S7(1)-118)
	Group member issues	Our group had a quarrel. (S7(3)-132) Every time I see him I feel very bad. (S2(2)-21) No one I know has a crying face. (S3(4)-48) The members of our group had a quarrel today! Just for a small puddle of water, I really do not understand (S7(2)-126)
Causes of negative deactivating emotion	Lecture-based teaching	Although I felt a little bored when the teacher talked about stress, the group interaction process was really interesting. (S1(3)-10) But I feel a little bored! (S7(3)-129)

**Table 6:** Coding results of reasons for differences in levels of agreement on stress relief effectiveness.

Parent Code	Child Code	Content Examples
High agreement	Effective stress relief methods	Most of the stress relief methods I learned here worked for me. (Q-S1(3)-1) I can learn how to truly relieve stress through design thinking. (Q-S2(1)-1) Stress relief designing can make all my stress disappear. I hope there will be courses like this in the future. (Q-S2(2)-1)
	Design thinking as a stress release	Sometimes you have to go on stage for presentation and you can watch other groups’ stress relief designs. If you use the normal class mode, it will be boring, so it is fun this way. (Q-S2(3)-1) Because after class, when my brother hits me at home, I do not want to hit him. (Q-S2(4)-1) I have relieved stress because I feel no pressure anymore. (Q-S3(1)-1) The class is rich in content and you can learn some knowledge without any pressure. (Q-S3(2)-1) Spending time with friends helps me relieve stress. (Q-S4(1)-1)
Low agreement	Classes as a source of stress	It cannot relieve stress. As long as you have to write down something or be graded, it has no stress-relieving function. (Q-S1(1)-1) I think although every activity is fun, I feel a little troublesome to write a lot of words. (Q-S1(2)-1) Because learning itself cannot relieve stress. (Q-S6(2)-1)

## Data Availability

The data presented in this study are available on request from the corresponding author.
